# Mutation analysis of the *WFS1* gene in a Chinese family with autosomal-dominant non-syndrome deafness

**DOI:** 10.1038/s41598-022-26850-3

**Published:** 2022-12-23

**Authors:** Jing Zhao, Siqi Zhang, Yuan Jiang, Yan Liu, Jiantao Wang, QingWen Zhu

**Affiliations:** 1 Department of Otolaryngology, The Third Hospital of Hebei Medical University, Hebei, China; 2 Department of Otolaryngology, The Second Hospital of Hebei Medical University, Hebei, China

**Keywords:** Genetics research, Disability, Mutation

## Abstract

To analyse the pathogenic genes and mutations of a family with hereditary deafness. We recruited a three-generation family with NSHL. A detailed medical history inquiry and related examinations were performed. Next-generation sequencing (NGS) was used to confirm the gene mutation in the proband, and Sanger sequencing was used for verification. The effect of the *WFS1* mutation on the function and structure of the wolframin protein was predicted by multiple computational software. From the Gene Expression Omnibus (GEO) database, we obtained GSE40585 dataset and performed enrichment analyses. The family clinically manifested as autosomal dominant NSHL. A novel *WFS1* c.2421C>G (p.Ser807Arg) mutation was identified in four affected individuals in the pedigree . The p.Ser807Arg mutation is a highly conserved residue and causes an increase in protein stability. It had an important influence on not only amino acid size, charge and hydrophobicity but also protein intermolecular hydrogen bonding and spatial structure. There were differentially expressed genes (DEGs) in GSE40585 dataset. Enrichment analysis revealed that DEGs mainly functioned in amino acid metabolism, signal transduction and dephosphorylation. We reported a novel mutation c.2421C>G (p.Ser807Arg in *WFS1*. This study expands the mutation spectrum of *WFS1*.

## Introduction

As one of the most common sensorineural defects, hereditary hearing loss is divided into syndromic hearing loss (SHL) and nonsyndromic hearing loss (NSHL), accounting for approximately 30% and 70% of HHL, respectively^1,2^. It is estimated that approximately 80% of NSHL cases are autosomal recessive, 15% are autosomal dominant, and less than 5% are X-linked/mitochondrial^3^. hereditary hearing loss has an extremely high degree of heterogeneity in terms of causative gene mutations and inheritance modes. To date, 51 causative genes and over 70 loci responsible for autosomal dominant nonsyndromic hearing loss (ADNSHL) have been confirmed (http://hereditaryhearingloss.org/; The Hereditary Hearing Loss Homepage). Nevertheless, there are still many mutations and genes involved in the onset and development of hearing loss unidentified. With the development of next-generation sequencing (NGS)^4^, characterized by high throughput and low cost, it is possible to screen all deafness-associated genes in a high-throughput manner. In our study, a Chinese ADNSHL family was recruited, and targeted deafness multigene sequencing and computational tools were used to explore the molecular basis of the mutation.

## Materials and methods

### Subjects

Proband with ADNSHL was recruited from the Second Hospital of Hebei Medical University. In addition to the proband, six additional members (I:2, II:2, II:3, II:5, II:7, III:3) of the family were recruited from the hospital, including two affected and three unaffected. The Ethics Committee of the Second Hospital of Hebei Medical University approved the study. We confirmed that all experiments were performed in accordance with relevant guidelines and regulations. We obtained informed consent from the subjects.

### Clinical information and examination

We obtained the medical histories of the subjects through a questionnaire on the following aspects: onset age, progression, tinnitus, noise exposure, use of ototoxic drugs, pregnancy/labour process, medication, use of hearing aids, and other relevant clinical manifestations. A detailed physical examination was performed in all patients and ruled out the possibility of syndromic hearing loss. The grades of hearing impairment were measured by pure tone audiometry (PTA). The average hearing threshold refers to the average values at 500, 1000, 2000, and 4000 Hz. According to the world report on hearing 2021 (https://www.who.int/publications/i/item/world-report-on-hearing), hearing levels were classified as normal (< 20 dB), mild (20 to < 35 dB), moderate (35 to < 50 dB), moderately severe (50 to < 65 dB), severe (65 to < 80 dB), profound (80 to < 95 dB), and complete (≥ 95 dB)^5^.

### Targeted sequencing and mutation analysis

For mutation analysis, peripheral blood (approximately 5–10 ml) was obtained from the subjects, and genomic DNA was extracted with a DNA extraction Kit (Qiagen, Shanghai, China) and stored at − 20 °C until use. We used the Nanodrop 2000 (Thermal Fisher Scientific, USA) to quantify the DNA and the GenCap® deafness gene system capture kit (MyGenostics Inc, Beijing, China) to capture the amplified DNA. The gene panel for hearing loss disease is summarized in Table S1. The capture experiment was conducted according to the manufacturer’s protocol. Targeted NGS of 415 known deafness genes was performed using the MyGenotics gene enrichment system and the HiSeq2000 sequencer (Illumina, USA). The parameter BWA of Sentieon software (https://www.sentieon.com/) can map the clean reads to the UCSC hg19 human reference genome. ANNOVAR software (http://annovar.openbioinformatics.org/en/latest/) annotated variants further and was associated with multiple databases, such as 1000 Genomes (http://www.1000genomes.org/), ESP6500, dbSNP, EXAC, Inhouse (MyGenostics), and HGMD. Computational programs, such as SIFT (http://sift.jcvi.org/), PolyPhen_2 (http://genetics.bwh.harvard.edu/pph2/), Mutation Taster (http://www.mutationtaster.org/), and GERP+, were used to predict the pathogenicity of the candidate variants. We used the guidelines of the American College of Medical Genetics and Genomics (ACMG) to interpret the data. Beijing MyGenostics Company performed the sequencing analysis.

### Mutation detection by Sanger sequencing

Validation primers were designed for the detected *WFS1* gene mutation sites, and Sanger sequencing was performed on subjects whose samples had been collected. Forward (5′-ACATCAAGAAGTTCGACCGC-3′) and reverse (5′- GCAATCTACACATGGTCGCA-3′) primers were designed using NCBI Primer-BLAST and synthesized by MyGenostics Company (Beijing, China). Sanger sequencing was used to verify the cosegregation of the pathogenic mutations and the hearing phenotype within the family.

### Mutation analysis by computational servers

MUpro (http://mupro.proteomics.ics.uci.edu), I-Mutant2.0 (https://folding.biofold.org/i-mutant/i-mutant2.0.html) and iStable (http://predictor.nchu.edu.tw/istable/indexSeq.php) were used to predict protein stability changes upon single point mutation. HOPE (http://www.cmbi.umcn.nl/hope) can analyse the impact of a given mutation on the protein structure. TMHMM Server 2.0 (https://services.healthtech.dtu.dk/service.php?TMHMM-2.0) is an online tool to predict transmembrane structures in proteins. Accessible Surface Area and Accessibility Calculation for Protein (ver. 1.2) online server can calculate solvent-accessible surface areas (SASA) of 890 amino acids of wolframin and S807 mutant protein. We built a three-dimensional (3D) model of the wolframin protein by Alphafold2 and presented the wild-type and mutant protein structures by PyMOL programs.

### Screening key genes and signalling pathways by bioinformatics analysis

GSE40585 gene expression profile microarray data were downloaded from GEO database (http://www.ncbi.nlm.nih.gov/geo/). The dataset was uploaded by Sulev Kõks in 2012 and included two types of HEK transfected cells: wild type and *WFS1* silenced type. Quantitative RT-PCR and western blot confirmed clear silencing of *WFS1* gene expression after 48 h. Therefore, we selected 4 samples transfected with blank plasmid for 48 h as the control group and 8 samples transfected with *WFS1* siRNA for 48 h as the experimental group. Using R software, we identified genes which were differentially expressed genes (DEGs) between groups. STRING (https://string-db.org/) was used to predict the protein–protein interaction (PPI) network of DEGs. Cytoscape software (v3.8.0) is used to visualize the network diagram. Gene Ontology (GO) and Kyoto Encyclopedia of Genes and Genomes (KEGG) pathway enrichment analyses were conducted on differential genes to investigate their potential biological functions. The Gene Set Enrichment Analysis (GSEA) (v.4.2.3) was performed for all genes in study samples.

## Results

### Clinical features

**Figure 1:**
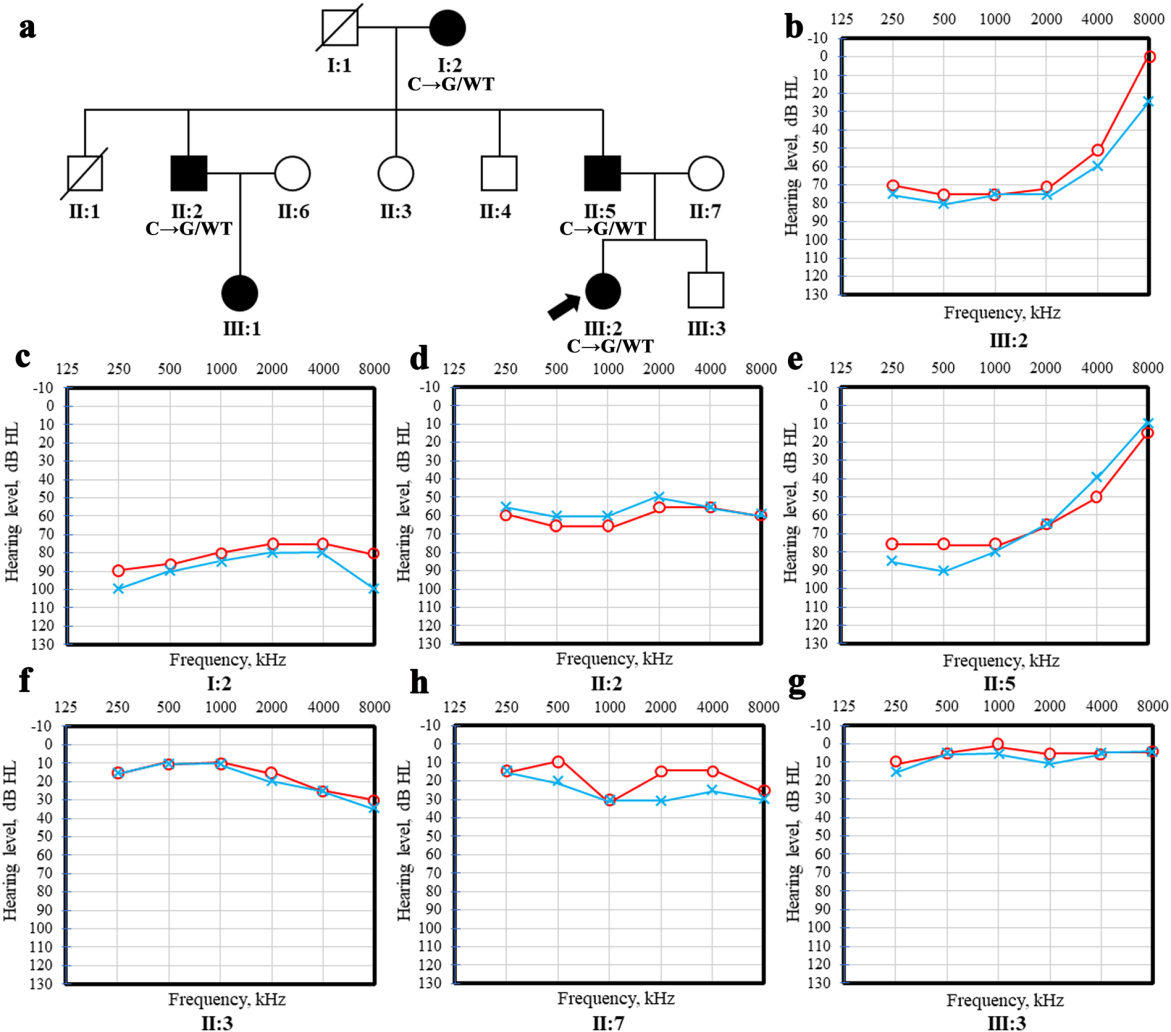
Combined figure. (**a**) Pedigreed diagrams of the family with autosomal dominant hearing loss (symbols with line, deceased). The affected subjects are denoted in black. The proband is indicated by an arrow. (**b**–**g**) Audiograms of the family subjects (red: right ear; blue: left ear). (**b**–**e**) Audiogram showed bilateral sensorineural hearing impairment of affected subjects. (**f–h**) tonal audiometric curves of unaffected family members.

There was a three-generation family with ADNSHL in China, including five affected patients and seven unaffected individuals. Seven people in the family participated in the research (4 affected and 3 unaffected). The affected member III:1 did not participate in the study due to personal reasons. The autosomal dominant inheritance pattern was shown in the family (Fig. 1a). None of the subjects in the study showed clinical signs in any other organs except hearing impairment. There was no vestibular or visual involvement. None of the members had ever used cochlear implants or hearing aids. Audiograms of the subjects of the family showed that their hearing impairments were bilateral, symmetric, sensorineural, postlingual, early onset and progressive (Table 1).

**Table 1:** Phenotypes and genotypes of the family subjects.

Member	Gender	Age at test (years)	Genotype		Phenotype			
			Exon8 (c.2421C > G)	Protein (p.S807R)	Age of Onset(years)	PTA (Right)(dB)	PTA (Left)(dB)	Level of hearing impairment
I:2	M	85	HT	➕	About 30	78.75	83.75	Severe
II:2	F	58	HT	➕	0–5	60.00	56.25	Moderately Severe
II:3	M	57	N	➖	–	N	N	Normal
II:5	F	48	HT	➕	0–5	66.25	68.75	Severe
II:7	M	49	N	➖	–	N	N	Normal
III:2	F	24	HT	➕	3	67.50	72.50	Severe
III:3	M	16	N	➖	–	N	N	Normal

F: female, M: male, mutation, ➖: no mutation, N: normal, HT heterozygous, HM homozygous, PTA: pure-tone audiometry.

A 24-year-old man is the proband, whose history of sensorineural hearing loss is 21 years. His disease is characterized by low frequency hearing loss, which gradually increased with age (Fig. 1b).The subject I:2 had an onset age in the third decade of life, which was the latest in this family, and her hearing impairment had slowly progressed to profound deafness with advancing age (Fig. 1c). The onset age of the subject II:2 was in the first decade of life, and his audiogram showed moderate hearing loss at all frequency (Fig. 1d). We could not get the exact age of onset of the subjects, except the proband, because of the negligence of their hearing loss and the lack of audiologic data at early ages. The subject II:5 had similar audiograms with the proband, but his left ear had a higher hearing threshold (Fig. 1e). The audiogram of the subjects II:3, II:7, III:3 were normal (Fig. 1f,g,h).

### Mutation detection and analysis

**Figure 2:**
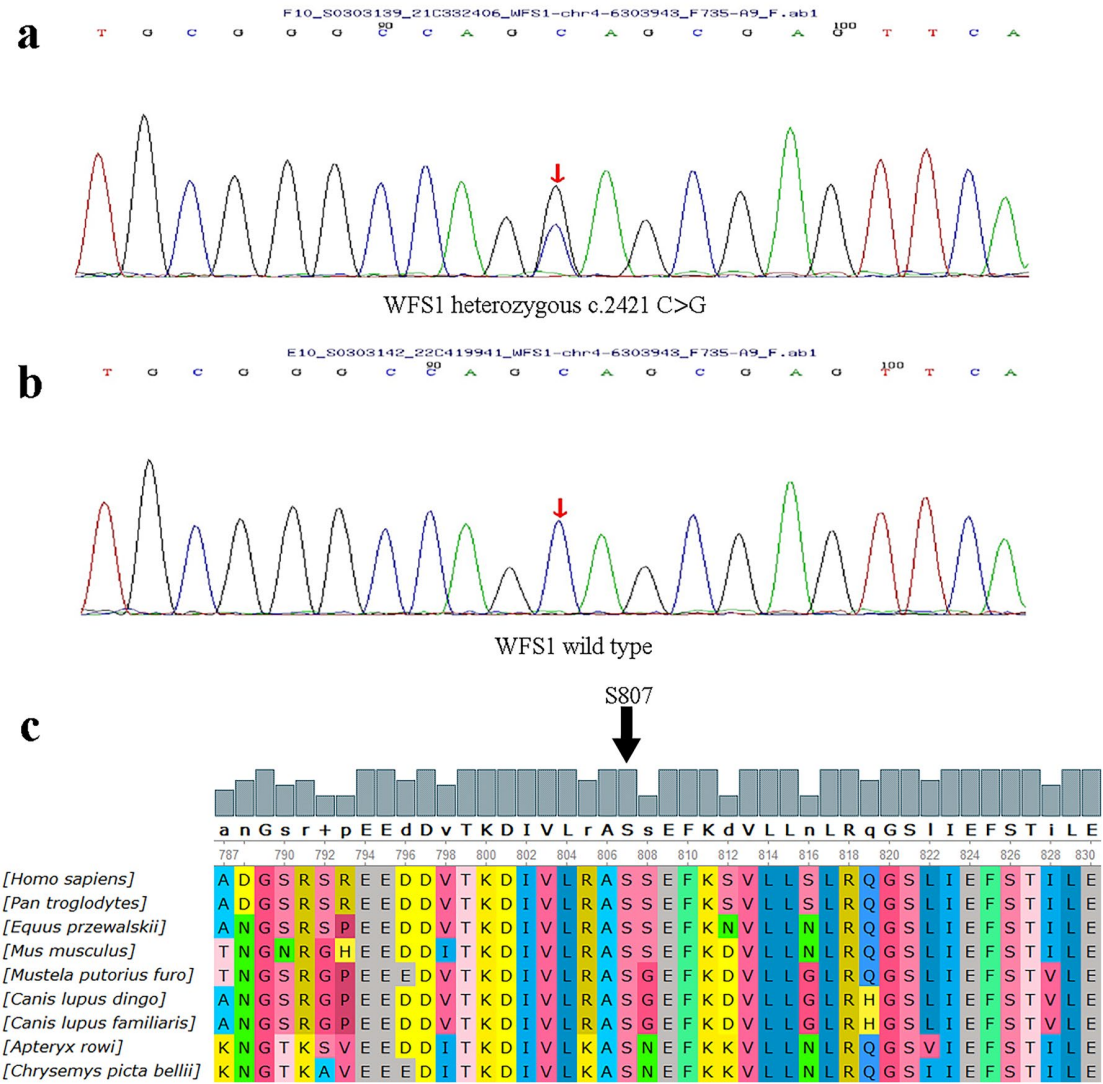
Mutation detection and conservation analysis. (**a**) Sequencing results show that the heterozygote c.2421C > G mutation was found in the proband (III:2). (**b**) unaffected family member (II:7). (**c**) Protein alignment shows conservation of the S807 residue of *WFS1* across 9 species.

A novel *WFS1* mutation in the Chinese ADNSHL family was identified in the study. The average sequencing depth for the targeted regions was 917.17-fold. There was 98.85% and 98.36% coverage of the targeted exons for 10X and 20X, respectively. In targeted sequencing, the c.2421C>G (p.Ser807Arg) mutation in the *WFS1* gene in exon 8 was identified in the proband (Fig. 2a). At amino acid 807, serine was replaced by arginine in the mutation. All patients carried this heterozygous mutation, but the normal family members did not carry the mutation (Fig. 2b). We verified the mutation by Sanger sequencing. This mutation cosegregated with hearing loss in four affected patients in pedigree (I:2; II:2; II:5; III:2) (Table 1). The prediction results using REVEL, SIFT, PolyPhen_2, Mutation Taster and GERP were shown in Table 2. All of the tools predicted that WFS1 c.2421C > G (p.Ser807Arg) was a damaging mutation. According to the ACMG guidelines ^6^, c.2421C>G (p.Ser807Arg) is classified as a likely pathogenic variant (PS1 + PM1 + PM2_Supporting + PP3). The mutation is classified as PS1, meaning that mutations of the same amino acid changes have been reported in database, but the nucleotide changes are different (Table 3). The p.Ser807Arg mutation localized in the intracytoplasmic hydrophilic C-terminal domain of the wolframin protein. The alignment of *WFS1* from various species is shown in Fig. 2c. It was shown that the serine residue at position 807 was highly conserved among different species, meaning it may play an important role in proper protein function.

**Table 2:** Pathogenicity assessment in silico of WFS1 c.2421C>G (p.Ser807Arg).

Tools	Pathogenicity	Functional prediction scores/conservation scores
REVEL	Deleterious	0.839
SIFT	Damaging	0.012
PloyPhen	Possibly_damaging	0.89
MutationTaster	Disease causing	1
GERP	Conserved	5.59

**Table 3:** Overview of all *WFS1* p.Ser807Arg mutations identified to date.

Number	Nucleotide change	Protein change	Exon	Domain	Origin	Audiometric configuration	References
1	c.2419A>C	p.Ser807Arg	Exon 8	C-terminal domain	United Kingdom	Low-frequency HL	Cryns et al.^32^
2	c.2419A>C	p.Ser807Arg	Exon 8	C-terminal domain	South Korea	Low-frequency HL	Choi et al.^33^
3	c.2421C>G	p.Ser807Arg	Exon 8	C-terminal domain	China	Low-frequency and flat HL	This study

### Computational analysis of the mutation effect

We further explored the changes in the secondary or tertiary structure of the protein that may be caused by the p.Ser807Arg mutation. According to the results of the ConSurf analysis, p.Ser807Arg had a conservation score of 9, meaning that it was highly conserved in evolution (Table 4). The effect of the p.Ser807Arg mutation on protein stability was predicted by MUpro, I-Mutant 2.0 and iStable software. It was shown that the p.Ser807Arg mutation resulted in increased protein stability. Compared with the wild type, the p.Ser807Arg mutation increased the size of the amino acid, changed the charge, and decreased the hydrophobicity. These changes may change the intramolecular interactions to influence the function of the wolframin protein.

**Table 4:** Prediction of p.Ser807Arg mutatant protein by using Mupro, I-Mutant 2.0(Seq), iStable and HOPE.

	Domain		Consurf score		Conservation	
Evolutionary conservation	C-terminal		9		Neither the mutant residue nor another residue type with similar properties was observed at this position in other homologous sequences. Based on conservation scores this mutation is probably damaging to the protein	

	MUpro		I-Mutant 2.0(Seq)		iStable	
	Confidence score	Prediction	DDG	Prediction	Confidence score	Prediction
Stability	0.31181111	Increase	− 0.1	Increase	0.843898	Increase

	Change of size		Change of charge		Change of Hydrophobicity	
HOPE	Mutant type>wild type		Neutral→positive		Decrease	
	The mutant residue is bigger, this might lead to bumps.		The mutation introduces a charge, this can cause repulsion of ligands or other residues with the same charge.		Hydrophobic interactions, either in the core of the protein or on the surface, will be lost.	

**Figure 3:**
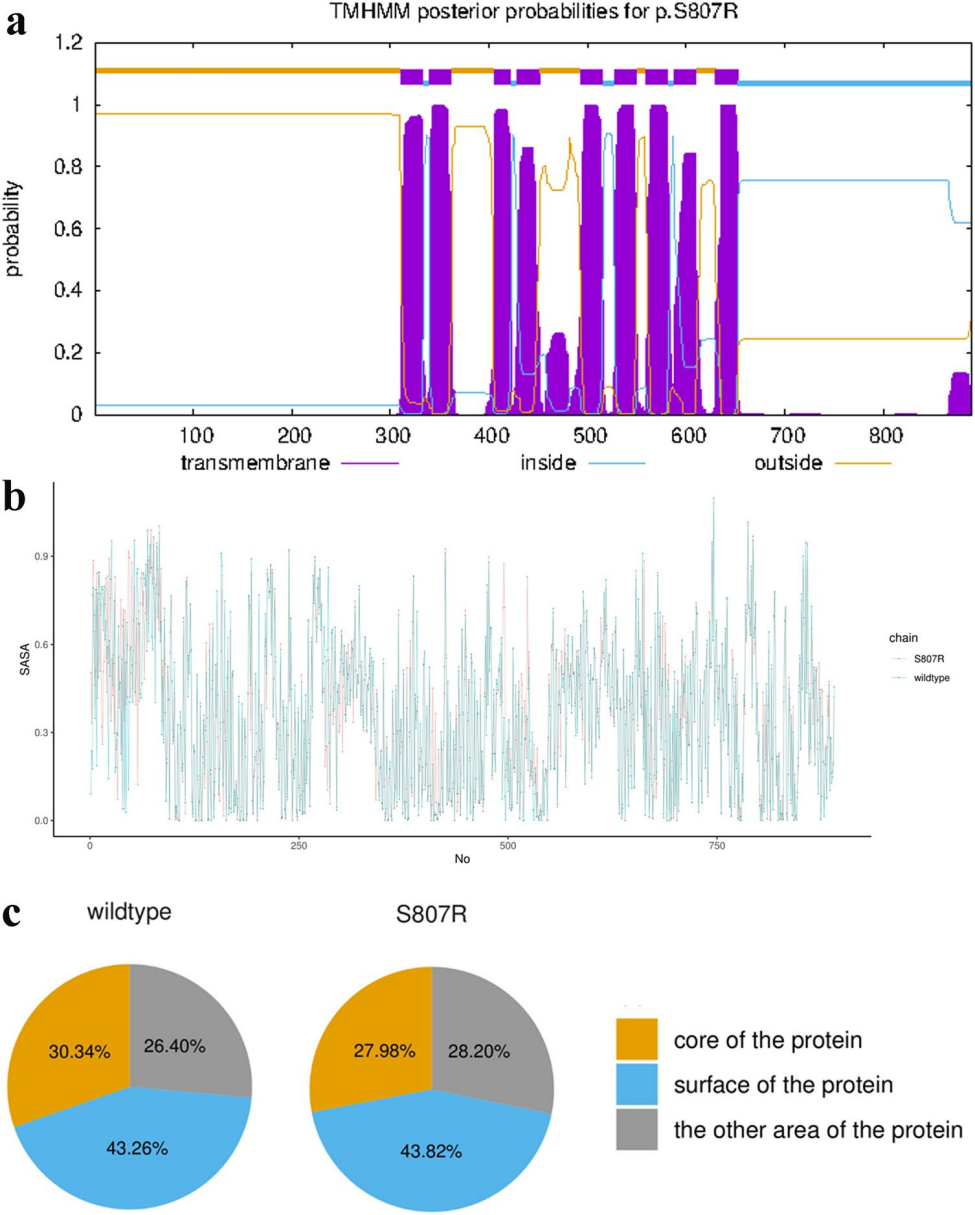
Combined figure. (**a**) The nine transmembrane regions in mutant S807R protein are shown by TMHMM server. (**b**) The bottom panels describe per-residue SASA of wolframin protein and mutant S807R protein. (**c**) The distribution about the SASA of all 890 amino acids in wild and mutant S807R protein.

We used TMHMM to investigate the effect of the p.Ser807Arg mutation on the transmembrane region of the wolframin protein. As shown in Fig. 3a, it was suggested that the p.Ser807Arg mutation resulted in no changes in the structure of the wolframin transmembrane region. According to SASA analysis, the residual fluctuations between the wild-type and mutant p.Ser807Arg proteins were similar (Fig. 3b). Compared with the wild type, the proportion of mutant p.Ser807Arg protein core amino acids was decreased, which suggested a detrimental influence on protein function (Fig. 3c).

**Figure 4:**
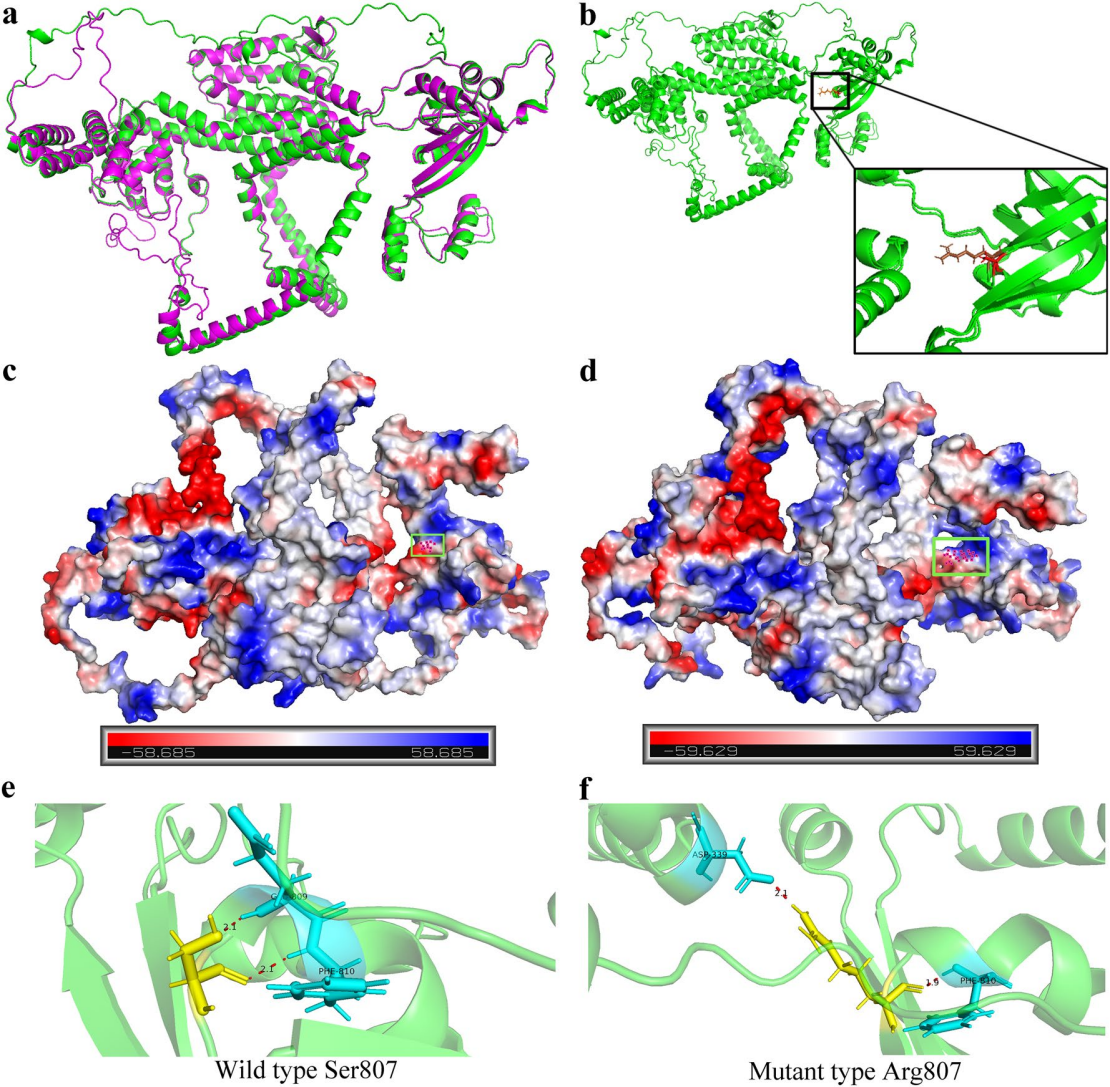
Mutation-induced structural changes in *WFS1*. (**a**) It shows superimposed view of wolframin protein in wild and mutant state. (**b**) Predicted structures depict the changes of mutant wolframin protein with the amino acid change S807R. Red and brown structures indicate differences between wild and mutant type. (**c**) A qualitative electrostatic representation of wolframin protein generated by PyMOL. Protein contact potentials can be represented by displaying virtual (false) red/blue charged smooth surfaces on wolframin protein. (**d**) A qualitative electrostatic representation of mutant S807R protein generated by PyMOL. The black circle indicates the position of amino acid 807. (**e–f**) The change of hydrogen bond between amino acids before and after mutation. The proteins are shown as cartoon. Amino acids at the mutated site 807 are highlighted in yellow and the interacting amino acids are highlighted in blue (red dotted line: the hydrogen bond).

The three-dimensional structures of wolframin and mutant p.Ser807Arg protein were predicted by the Robetta server. To compare the three-dimensional structure before and after mutation, the p.Ser807Arg mutant protein was superimposed on the wild-type protein by PyMOL (Fig. 4a). The substitution regions were highlighted in the protein model (Fig. 4b). Comparing the qualitative electrostatic representation between the wild-type and mutant p.Ser807Arg proteins, the p.Ser807Arg mutation changed the charge of the amino acid at this site from neutral to positive (Fig. 4c,d). In the wild type, Ser807 had a hydrogen bond with Glu809 and Phe810 (Fig. 4e). In the mutant, the original hydrogen bond distances between Arg807 and Phe810 became shorter, while the hydrogen bond between Arg807 and Glu809 disappeared (Fig. 4f). In addition, a hydrogen bond between Arg807 and Asp339 was added. The stability and intramolecular interactions of the wolframin protein may be affected by the above changes in hydrogen bonds, resulting in diseases.

### Hub genes screening and enrichment analysis

**Figure 5:**
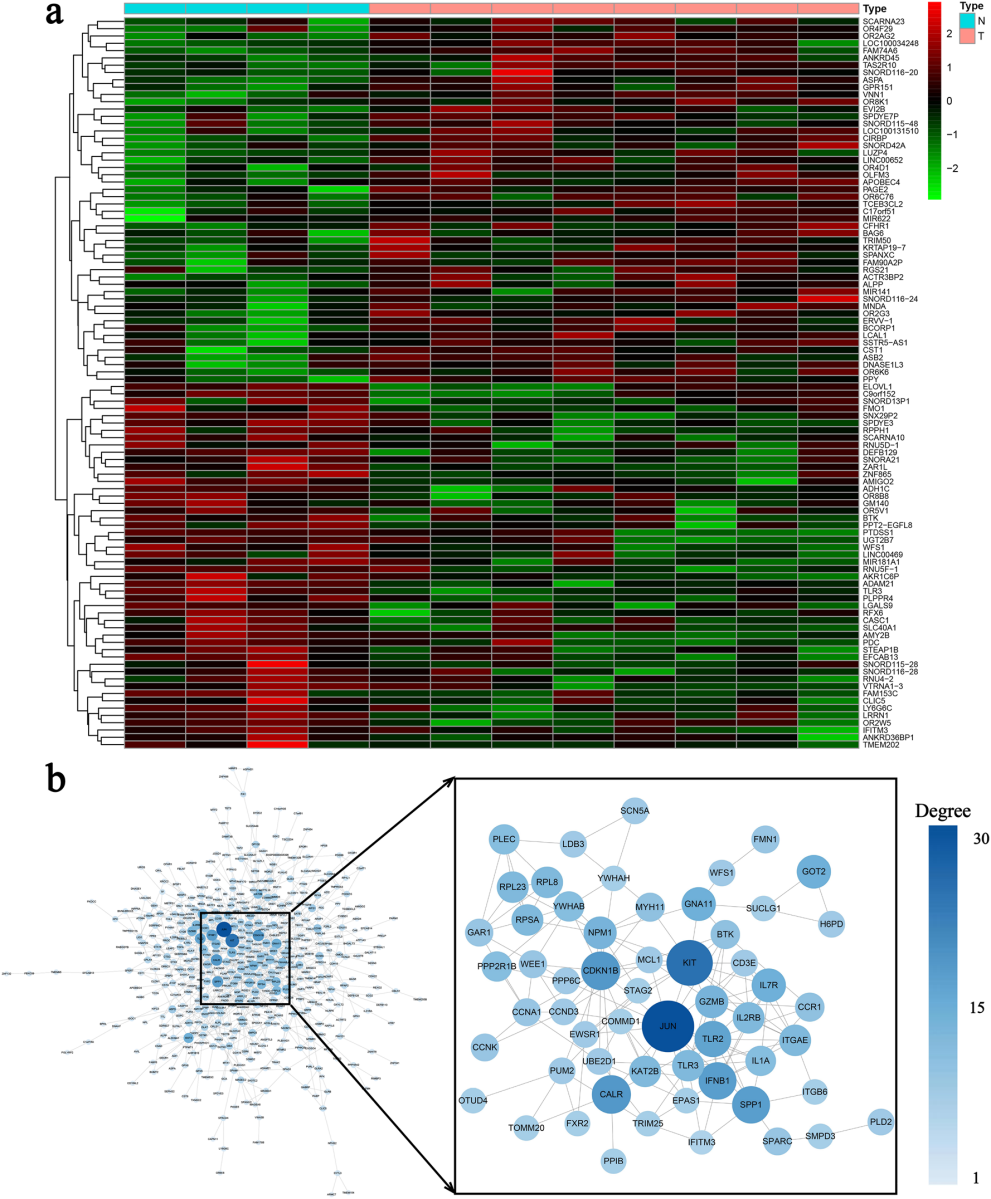
Heat map and PPI network. (**a**) Heat map of the 12 samples. Each column represents a sample, and each row represents one of the differentially expressed genes. (**b**) PPI network of GSE40585.

**Figure 6:**
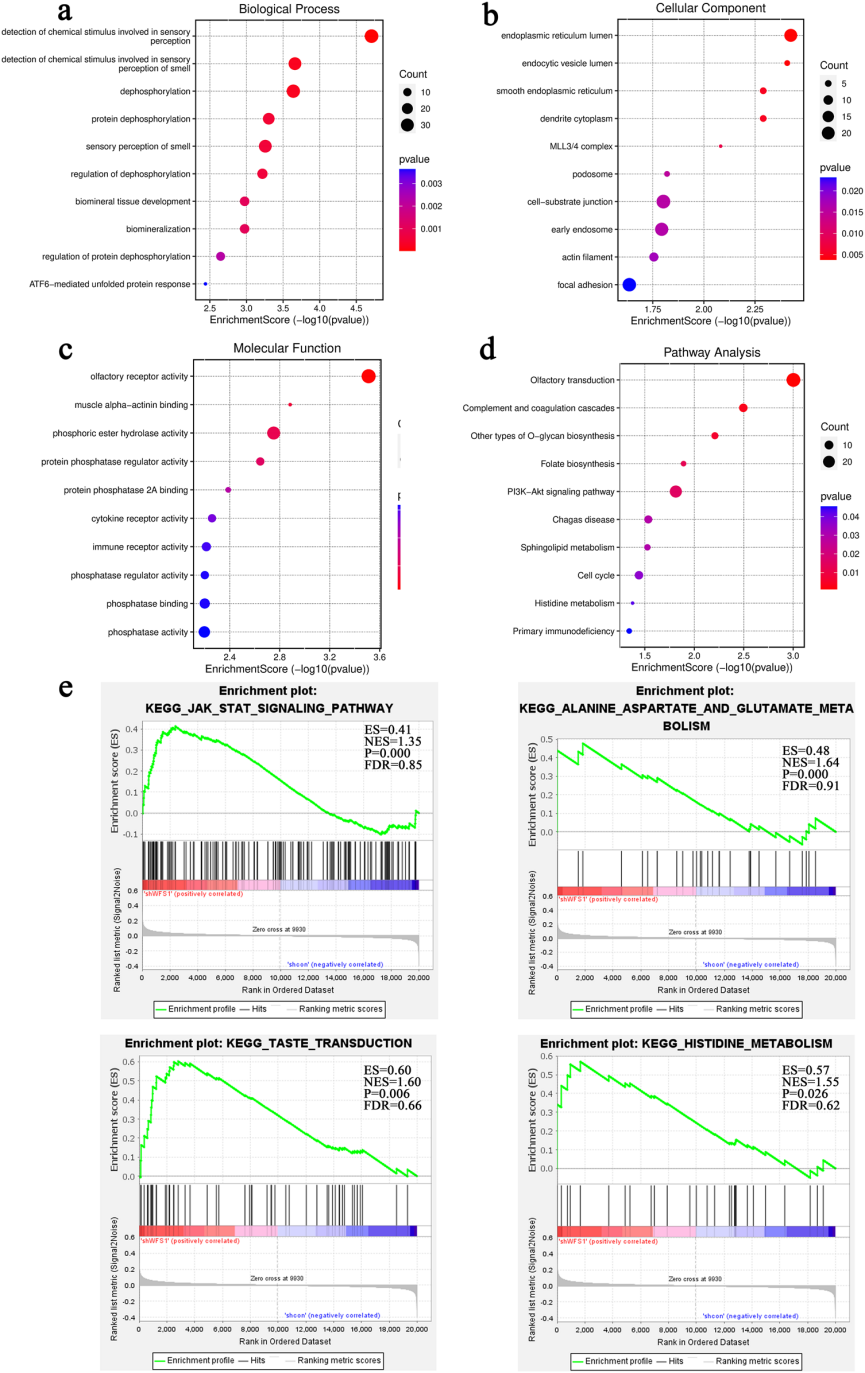
Enrichment analysis between wild type and *WFS1* silenced type. (**a**) Biological process of DEGs. (**b**) Cellular component of DEGs. (**c**) Molecular function of DEGs. (**d**) KEGG pathway of DEGs. (**e**) Significant KEGG enrichment plot from GSEA analysis.

For the purpose of exploring the potential mechanism of deafness caused by *WFS1* gene mutation, we have screened key genes and signaling pathways by integrated bioinformatics analysis. A heatmap of DEGs is shown in Fig. 5a, while a PPI network is shown in Fig. 5b. Jun proto-oncogene (*JUN*), KIT proto-oncogene (*KIT*), cyclin dependent kinase inhibitor 1B(*CDKN1B*), calreticulin (*CALR*), interferon beta 1(*IFNB1*), sapiens secreted phosphoprotein 1(*SPP1*) and Toll Like Receptor 2 (*TLR2*) were indicated as hub genes. According to GO analysis, the changes of biological process (BP) of DESs were mainly concentrated in detection of chemical stimulus involved in sensory perception and dephosphorylation (Fig. 6a–c). KEGG pathway analysis showed that olfactory transduction, complement and coagulation cascades and other types of o-glycan biosynthesis were statistically significant (Fig. 6d). Comparing gene expression differences between groups is the purpose of GO and KEGG enrichment analysis. GSEA analyses the whole gene dataset, so genes with little differential expression but a great deal of significance will not be excluded from GSEA analysis (Fig. 6e). JAK-STAT signalling pathway, amino acid metabolism, and taste transduction are the most statistically significant biological processes.

## Discussion

The *WFS1* gene, also named Wolfram syndrome type 1, encodes the wolframin protein. It is a membrane glycoprotein localized in the endoplasmic reticulum (ER). Wolframin has been shown to be highly expressed in the pancreas, brain, heart, and muscle but expressed differentially in inner ear cells^7^. It plays a critical role not only in the regulation of insulin production and secretion from pancreatic β-cells but also in the regulation of ER stress response and cellular calcium homeostasis^8^. Wolframin can negatively regulate the ER stress signalling network to avoid the accumulation of misfolded and unfolded proteins^9^. Wolframin can modulate the filling state of the ER Ca^2+^ store to participate in the regulation of cellular Ca^2+^ homeostasis^10^. Once a variant occurs in *WFS1*, ER stress is strongly induced, and endolymphatic ion composition and homeostasis are disrupted, which leads to deafness.

There are more than 490 reported mutations of the *WFS1* gene in the Human Gene Mutation Database. Mutations in the *WFS1* gene are predominantly responsible for nonsyndromic hearing loss (DFNA6), Wolfram syndrome and Wolfram-like syndrome ^11,12^. In recent years, some studies have also shown that *WFS1* is linked to type 2 diabetes, autosomal dominant optic atrophy (ADOA), and psychiatric problems^13–15^. *WFS1*-associated Wolfram syndrome type 1 (WS1) is defined as a rare autosomal neurodegenerative recessive progressive disease and is also known as DIDMOAD, characterized by diabetes insipidus (DI), childhood onset diabetes mellitus (DM), optic atrophy (OA), and deafness (D)^16^. Wolfram-like syndrome is an uncommon autosomal dominant disease that contains variable clinical manifestations, including sensorineural hearing loss, optic atrophy and/or diabetes mellitus^17^. The *WFS1*-associated nonsyndromic hearing loss has two types: autosomal dominant hearing loss, characterized by low-frequency decline, and autosomal recessive hearing loss, characterized by congenital profound sensorineural deafness.

Low-frequency sensorineural hearing loss (LFSNHL) is a rare form of hearing loss that affects only 2000 Hz and below frequencies and generally deteriorates over time without progressing to severe deafness^18^. Only two associated genes have been reported to date: DIAPH1 and *WFS1*^19,20^. DIAPH1 is linked to the DFNA1 locus on chromosome 5q31^21^. It is characterized by rapid progression, eventually developing into profound deafness, from low to involve all higher frequencies. DFNA54 is the third locus reported to be associated with LFSNHL. It was mapped in a large Swiss family harbouring an unknown gene that results in low frequency hearing impairment when affected^22^. In 2001, mutations in the *WFS1* gene were confirmed to be one of the causes of LFSNHL, which was the causative gene at the DFNA6 and DFNA14 loci^23^. In the same year, Young et al. came to the same conclusion through linkage analysis of a 6-generation LFSNHL family in Canada, thus suggesting that the causative genes of DFNA6, DFNA14 and DFNA38 are the same—the *WFS1* gene^24^. Generally, ADNSHL patients with *WFS1* mutations mostly have progressive, bilateral sensorineural hearing loss. In this study, the grandmother and uncle of the proband (I:2 and II:2) besides low frequency sensorineural hearing impairment, also show impairment at higher frequencies. It may be related to ageing in the grandmother and to working in a noisy environment for a long time in the uncle.

*WFS1*-accociated hearing loss can be caused by in-frame insertions or deletions, multiple protein terminating mutations, missense mutations and so on^25–27^. We summarized all types of mutations in the *WFS1* gene that have been identified thus far in NSHL from the Human Gene Mutation Database (Tables S1–S2)^28–31^. In fact, the most common mutations associated with LFNSHL are missense mutations located in exon 8. In this study, we found that the *WFS1* gene c.2421C>G (p.Ser807Arg) heterozygous mutation, located in exon 8, is the gene that causes deafness in this Chinese family. There was no report about the novel mutation in public databases. However, a mutation at the same residue (p.Ser807Arg) causing LFSNHL was first found in an English family^32^. Then, it was also reported in a Korean family^33^. The c.2419A>C (p.Ser807Arg) mutation was found in the Korean family, and the affected members had symmetrical bilateral hearing loss, which was mild to moderate in severity. The c.2421C>G (p.Ser807Arg) mutation in a Chinese family was first reported and identified in this study. The analysis of p.Ser807Arg mutations in the *WFS1* gene is shown in Table 3. Missense mutations normally partially destroy the function of wolframin protein^7^. However, its exact physiological role in the cochlea remains unknown.

Generally, the majority of NSHL patients with mutations in *WFS1* experienced hearing loss change from low frequency to full frequency, with no or slow progression except for presbycusis. Its onset time varies greatly from prelingual to the early 40 s. Most of them are heterozygous missense mutations, which belong to autosomal dominant inheritance. However, the homozygous variant c.972C>G of *WFS1* in an Egyptian family was reported. The affected members presented with congenital bilateral severe to severe hearing loss, but they did not have other manifestations of Wolfram syndrome, presenting autosomal recessive inheritance ^34^. The most common hearing loss is the low frequency type, but some studies have proven that *WFS1* mutation can also lead to middle, high or all frequency hearing loss; for example, Chinese researchers have reported that the mutation c.2389G>A (p. Asp797Asn) causes NSHL at all frequencies^35^. Some studies have shown that *WFS1* mutations also play an important role in age-related hearing loss (ARHL). For example, a pathogenic mutation 1582T>C (p. Tyr528His) in *WFS1* was identified in an ARHL Finnish family^36^. It is suggested that the wolframin protein could participate in the signalling pathway of the unfolded protein response (UPR)^37^, which is related to the pathogenesis of ARHL^38^.

To date, it has been reported that at least 71 different missense mutations in *WFS1* are associated with NSHL, comprising 65 mutations in exon 8, four in exon 5, and one in exon 4 (Table S1). The mutations associated with NSHL were mainly concentrated in exon 8 and mostly clustered in the C-terminal domain of the wolframin protein (amino acids 652–890), suggesting that the region was very important for proper protein function of the inner ear. The mutations associated with DIDMOAD mostly do not cluster in any particular region of the protein and tend to be inactivating, which means that the cause of Wolfram syndrome is probably a loss of function of the wolframin protein. However, the causative mutations were found to be non-inactivating in NSHL, which suggests that NSHL results from specific mutations that partially destroy the function of intact proteins. The C-terminal region of wolframin is located on the cytoplasmic side of the ER membrane, adopting a folded confirmation. It can interact with the C-terminal domain of the ER-localized Na+/K+ ATPase beta1 subunit, which is important for subunit maturation^39^. To our knowledge, Na+/K+ ATPase deficiency is responsible for apoptosis and neural degenerative disease. If there are similar associations in the inner ear, amino acid substitutions may result in hearing loss in this way. It is also predicted that amino acid substitutions could change the secondary structure by disrupting helical chains, interfering with the ability of the C-terminal region to interact with other proteins in the ER lumen^40^. In conclusion, missense mutations in *WFS1* may act in a dominant-negative way, causing hearing loss by disrupting protein‒protein interactions or protein phosphorylation.

Several signalling pathways may be upregulated or downregulated when the *WFS1* gene is knocked down. The PPI network and hub genes analysis indicated that the knockdown of *WFS1* had significant influence on carcinogenesis and development of cancer, calcium regulation, signal transduction, dephosphorylation. The JAK/STAT pathway is involved in lots of biological processes, such as immune regulation, cell division and apoptosis, and also plays a critical role on the embryonic development of the inner ear^41^. There are a number of amino acids that can act as neurotransmitters or neuromodulators, including alanine, aspartate, glutamate, and histidine^42^. *WFS1* mutation may cause significant interference in amino acid metabolism pathway involved in neurotransmission. In addition, histidine may be related to metabolic syndrome and affect ion absorption^43^. All the analysis provided an indication for further study of the deafness mechanism caused by *WFS1* mutation.

In conclusion, our study showed that the c.2421C>G p.Ser807Arg) mutation is a novel mutation of the *WFS1* gene in the Chinese population. We recommended that *WFS1* gene testing should be incorporated into routine examination for the genetic diagnosis of deafness, especially low-frequency hearing loss. Low-frequency hearing loss usually cannot be identified by routine methods of newborn hearing screening^44^, so it is necessary for LFNSHL families to conduct genetic counselling and genetic testing.

## Supplementary Information

Supplementary Table S1.

Supplementary Table S2.

Supplementary Information 3.

## Data Availability

The data supporting the results reported in the article can be found in the Supplementary Information files. The datasets generated and/or analysed during the current study are available in the NCBI database with the accession number SUB12158042 (https://www.ncbi.nlm.nih.gov/).
