# Supplementary material for: Mutation analysis of the WFS1 gene in a Chinese family with autosomal-dominant non-syndrome deafness
Source: Sci Rep. 2022 Dec 23;12:22180. doi: 10.1038/s41598-022-26850-3 (PMC9789122; doi:10.1038/s41598-022-26850-3)
Supplement: Supplementary file 1 — Supplementary Table S1. [file 41598_2022_26850_MOESM1_ESM.docx]

**Supplementary Table S1. Summary of missense mutations in WFS1 associated with NSHL from previous studies**

| **Number** | **Nucleotide change** | **Amino acid change** | **Exon** | **Domain** | **Hereditary Pattern** | **Age of onset** | **Audiometric configuration** | **Hearing level** | **ACMG classification** | **Origin** | **Reference** |
| --- | --- | --- | --- | --- | --- | --- | --- | --- | --- | --- | --- |
| 1 | 353A>C | Asp118Ala |  |  | AR |  |  |  | Likely pathogenic | Western Europe | Manou Sommen et al.2016 |
| 2 | 449C>T | Ala150Val | 4 | N-terminal | AD | congenital | HF | severe to profound |  | China | Jie Qing et al.2014 |
| 3 | 482G>A | Arg161Gln | 5 | N-terminal |  |  | LF |  |  |  | Barrett et al. 2009 |
| 4 | 511G>A | Asp171Asn | 5 | N-terminal | AD | third decade of life | LF | moderate |  | Portugal | A.C.Gonçalves et al. 2014 |
| 5 | 559C>T | Leu187Phe | 5 | N-terminal | AD | congenital |  | severe to profound | VUS | Iran | Javad Mohammadi-Asl et al.2021 |
| 6 | 577A>C | Lys193Gln | 5 | N-terminal | Sporadic | early onset | LF |  |  | Germany | Kim Cryns et al.2002 |
| 7 | 908T>C | Leu303Pro | 8 | N-terminal | AD |  | LF MF | mild to moderate | Likely Pathogenic | Japan | Masafumi Kobayashi et al.2018 |
| 8 | 923C>G | Ser308Cys | 8 | N-terminal | AD | 6;16 | LF MF | moderate | Likely Pathogenic | Japan | Masafumi Kobayashi et al.2018 |
| 9 | 972C>G | Ile324Met | 8 | TM1 | AR | congenital |  | severe to profound |  | Egypt | Birgit S Budde et al.2020 |
| 10 | 1015G>A | Asp339Asn | 8 |  |  |  | LF HF |  | VUS | Europe/United States/Japan | W. Daniel Walls et al.2020 |
| 11 | 1072G>A | Val358Met | 8 | TM2 | AR | postlingual |  | severe to profound |  | Iran | Christina M Sloan-Heggen et al. 2015 |
| 12 | 1147C>T | Arg383Cys | 8 | Cytoplasm Between TM2 and TM3 |  |  | Flat | moderate | Likely Pathogenic | Japan | Yoh-ichiro Iwasa et al.2016 |
| 13 | 1235T>C | Val412Ala | 8 | TM3 | AD |  | LF HF | mild |  | Korea | Byung Yoon Choi et al. 2013 |
| 14 | 1264G>T | Ala422Ser | 8 |  |  |  | LF HF |  | VUS | Europe/United States/Japan | W. Daniel Walls et al.2020 |
| 15 | 1309G>A | Gly437Ser | 8 |  |  |  |  |  |  | China | Siqi Chen et al.2016 |
| 16 | 1371G>T | Arg457Ser | 8 |  |  |  | LF |  |  |  | Smith et al. 2004 |
| 17 | 1480G>A | Gly494Ser | 8 | TM5 | AD (de novo) | 0 | LF | mild |  | Japan | Natsuko Kasakura-Kimura et al.2017 |
| 18 | 1554G>A | Met518Ile | 8 |  |  |  | LF |  |  |  | Smith et al. 2004 |
| 19 | 1582T>C | Tyr528His | 8 |  | AD | >40 | MF HF | moderate to severe |  | Finland | Laura Kytövuori et al.2017 |
| 20 | 1669C>T | Leu557Phe | 8 | TM7 |  |  | LF |  | VUS |  | Smith et al. 2004 |
| 21 | 1805C>T | Ala602Val | 8 | TM8 |  |  | LF |  |  |  | Smith et al. 2004 |
| 22 | 1820C>T | Pro607Leu | 8 | TM8 | Sporadic | childhood |  | mild to moderate | Pathogenic | United States | Christina M Sloan-Heggen et al. 2016 |
| 23 | 1831C>T | Arg611Cys | 8 |  | AD | 11~20 |  |  | Pathogenic | United States | Christina M Sloan-Heggen et al. 2016 |
| 24 | 1846G>T | Ala616Ser | 8 | Cytoplasm Between TM8 and TM9 |  |  | LF |  | VUS | China | Liu et al. 2005 |
| 25 | 1871T>C | Val624Ala | 8 | Cytoplasm Between TM8 and TM9 | AD | 5~14 | LF |  |  | Japan | Hisakuni Fukuoka et al.2007 |
| 26 | 1901A>C | Lys634Thr | 8 | TM9 | AD | <17 | LF | moderate |  | Japan | Kazuki Komatsu et al. 2002 |
| 27 | 1957C>T | Arg653Cys | 8 | C-terminal |  |  | LF | midl to moderate |  | China | Wei et al. 2014 |
| 28 | 1982A>G | Asn661Ser | 8 | C-terminal | AD | 6 | LF | moderate | VUS | Japan | Masafumi Kobayashi et al.2018 |
| 29 | 2005T>C | Tyr669His | 8 | C-terminal | AD | <22 | LF | moderate |  | Taiwan China | Hsun-Tien Tsai et al.2007 |
| 30 | 2020G>T | Gly674Trp | 8 | C-terminal | AD | 19~30 | LF | mild to profound | Pathogenic | China | Jinying Li et al.2021 |
| 31 | 2021G>A | Gly674Glu | 8 | C-terminal | AD | 0 | LF | moderate | Likely Pathogenic | Netherlands | Kim Cryns et al.2002 |
| 32 | 2021G>T | Gly674Val | 8 | C-terminal | AD | 0 | LF | moderate | Pathogenic | Netherlands | Kim Cryns et al.2002 |
| 33 | 2027G>A | Arg676His | 8 | C-terminal | AD |  |  |  | Likely Pathogenic | Japan | Hideaki Moteki et al.2015 |
| 34 | 2029G>A | Ala677Thr | 8 | C-terminal | AD | 0~10 |  |  | Pathogenic | United States | Christina M Sloan-Heggen et al. 2016 |
| 35 | 2032T>C | Trp678Arg | 8 | C-terminal | AD |  | MF |  |  | Netherlands | Celia Zazo Seco et al.2017 |
| 36 | 2033G>T | Trp678Leu | 8 | C-terminal |  |  | LF |  |  | United States | Sivakumaran et al.2004 |
| 37 | 2045A>G | Asn682Ser | 8 | C-terminal | Sporadic | 4 | LF | moderate | VUS | Japan | Masafumi Kobayashi et al.2018 |
| 38 | 2054G>C | Arg685Pro | 8 | C-terminal | AD |  | LF | moderate to severe | Likely Pathogenic | United States | Naomi F Bramhall et al.2008 |
| 39 | 2086C>T | His696Tyr | 8 | C-terminal | AD | 5~28 | LF Flat | mild to severe |  | China | Yi Sun et al. 2011 |
| 40 | 2096C>T | Thr699Met | 8 | C-terminal |  | <25 | LF | moderate | Pathogenic | Netherlands | I N Bespalova et al. 2001 |
| 41 | 2108G>A | Arg703His | 8 | C-terminal | Sporadic |  | LF |  |  | China | Yi Sun et al. 2011 |
| 42 | 2115G>C | Lys705Asn | 8 | C-terminal |  | 0 | LF | moderate | Pathogenic | Germany | Kunz et al. 2003 |
| 43 | 2119G>T | Val707Phe | 8 | C-terminal |  |  |  |  | Likely Pathogenic | United States | W. Daniel Walls et al.2020 |
| 44 | 2141A>T | Asn714Ile | 8 | C-terminal | AD | childhood |  |  | Pathogenic | United States | Christina M Sloan-Heggen et al. 2016 |
| 45 | 2146G>A | Ala716Thr | 8 | C-terminal |  | <10 | LF | moderate to severe | Pathogenic | United States | I N Bespalova et al. 2001 |
| 46 | 2209G>A | Glu737Lys | 8 | C-terminal |  |  |  |  |  | China | Liu et al. 2005 |
| 47 | 2272A>G | Lys758Glu | 8 | C-terminal |  |  | LF HF |  | VUS | Europe/United States/Japan | W. Daniel Walls et al.2020 |
| 48 | 2282C>T | Ala761Val | 8 | C-terminal | AD | congenital |  |  | Pathogenic | United States | Christina M Sloan-Heggen et al. 2016 |
| 49 | 2311G>C | Asp771His | 8 | C-terminal | AD | 5~20 | LF Flat | moderate to severe | Pathogenic | Switzerland | Nicolas Gürtler et al. 2005 |
| 50 | 2321A>C | Lys774Thr | 8 | C-terminal | AR | postlingual |  | severe to profound |  | Iran | Christina M Sloan-Heggen et al. 2015 |
| 51 | 2335G>A | Val779Met | 8 | C-terminal |  |  | LF |  |  | United States | I N Bespalova et al. 2001 |
| 52 | 2359G>A | Ala787Thr | 8 | C-terminal |  |  | Flat | profound |  | Japan | Yoh-ichiro Iwasa et al.2016 |
| 53 | 2389G>A | Asp797Asn | 8 | C-terminal | AD | 1~17 | Flat HF | severe to profound |  | China | Xiaohui Bai et al.2014 |
| 54 | 2419A>C | Ser807Arg | 8 | C-terminal | AD | early onset | LF |  |  | United Kingdom | Kim Cryns et al.2002 |
| 55 | 2437G>A | Val813Met | 8 | C-terminal | sporadic | prelingual |  | Moderately severe | VUS | Slovenia / BiH | Tina Likar et al.2018 |
| 56 | 2486T>C | Leu829Pro | 8 | C-terminal |  | 6~32 | LF | moderate | Pathogenic | United States | I N Bespalova et al. 2001 |
| 57 | 2491G>A | Gly831Ser | 8 | C-terminal | AD (de novo) | 1 | Flat | profound |  | Finland | Sanna Häkli et al.2014 |
| 58 | 2492G>A | Gly831Asp | 8 | C-terminal | AD | <20 | LF | moderate | Pathogenic | United States | Kim Cryns et al.2002 |
| 59 | 2501G>A | Gly834Asp | 8 | C-terminal |  | early/adult | LF HF | moderate |  | Italy | [Anna Morgan et al.2018](https://www.ncbi.nlm.nih.gov/pubmed/?term=Morgan%20A%5BAuthor%5D&cauthor=true&cauthor_uid=30622556) |
| 60 | 2507A>C | Lys836Thr | 8 | C-terminal | AD | 2~10 | MF LF | moderate | Pathogenic | Japan | Taro Fujikawa et al. 2010 |
| 61 | 2530G>A | Ala844Thr | 8 | C-terminal | AD | 6 | LF | moderate |  | Japan | Yoshihiro Noguchi et al.2005 |
| 62 | 2567C>A | Pro856His | 8 | C-terminal | AD |  |  |  |  | Italy | Anna Morgan et al.2020 |
| 63 | 2576G>A | Arg859Gln | 8 | C-terminal | AD | 2~45 | LF MF | mild to moderate |  | United States | Michael S. Hildebrand et al.2008 |
| 64 | 2576G>C | Arg859Pro | 8 | C-terminal | AD | 5~30 | LF Flat | moderate | Likely Pathogenic | United States | Nicolas Gürtler et al. 2005 |
| 65 | 2576G>C | Arg859Gln | 8 | C-terminal |  | 2~45 | LF | moderate | Likely Pathogenic | United States | Hildebrand et al.2008 |
| 66 | 2590G>A | Glu864Lys | 8 | C-terminal |  | 4 | LF | moderate to severe | Pathogenic | Japan | Hisakumi Fukuoka et al. 2008 |
| 67 | 2591A>G | Glu864Gly | 8 | C-terminal | AD | childhood;17;48 | LF Flat | mild to profound | Pathogenic | China | Zhijie Niu et al.2018 |
| 68 | 2596G>A | Asp886Asn | 8 | C-terminal |  |  |  |  |  | China | Liu et al. 2005 |
| 69 | 2603G>A | Arg868His | 8 | C-terminal | Sporadic | childhood |  | mild to moderate | Pathogenic | United States | Christina M Sloan-Heggen et al. 2016 |
| 70 | 2645T>C | Phe882Ser | 8 | C-terminal |  |  | LF HF |  | VUS | Europe/United States/Japan | W. Daniel Walls et al.2020 |
| 71 | 2663C>T | Ser888Leu | 8 | C-terminal | AR |  |  |  | Likely pathogenic | Western Europe | Manou Sommen et al.2016 |

TM: transmembrane; AR: autosomal recessive; AD: autosomal dominant; LF: low frequency; MF: middle frequency; HF: high frequency; VUS: variant of unknown clinical significance.
