# Supplementary material for: Mutation analysis of the WFS1 gene in a Chinese family with autosomal-dominant non-syndrome deafness
Source: Sci Rep. 2022 Dec 23;12:22180. doi: 10.1038/s41598-022-26850-3 (PMC9789122; doi:10.1038/s41598-022-26850-3)
Supplement: Supplementary file 2 — Supplementary Table S2. [file 41598_2022_26850_MOESM2_ESM.docx]

**Supplementary Table S2. Summary of all reported variants (except missense mutation) in WFS1 associated with NSHL from previous studies**

| **Number** | **Reference Nucleotide** | **Nucleotide Change** | **HGVS Variant Description** | **Protein Change** | **Exon** | **Domain** | **Hereditary Pattern** | **Age of onset** | **Audiometric configuration** | **Hearing level** | **ACMG classification** | **Origin** | **Reference** |
| --- | --- | --- | --- | --- | --- | --- | --- | --- | --- | --- | --- | --- | --- |
| 1 | T | TTCATCACCGTGC | 1463_1474dupTCATCACCGTGC | ins 12 bp codon 492 |  |  | AD | postlingual | LF Flat | profound to severe | VUS | Spain | Gema García-García et al.2020 |
| 2 | AAGG | A | c.2036_2038delAGG | del 3 bp codon 680 | 8 | c-terminal | AD | late onset | LF | mild to moderate |  | China | Qinjun Wei et al.2014 |
| 3 | TCGA | **T** | 2137_2139delGAC | del 3 bp codon 713 | 8 | c-terminal | AD | congenital |  |  |  | United States | Christina M Sloan-Heggen et al. 2016 |
| 4 | G | GA | 2150dupA | ins 1 bp codon 717 |  |  | Sporadic | >40 | HF |  |  | France | Sophie Boucher et al.2020 |
| 5 | ACAT | A | 2300_2302delTCA | del 3 bp codon 767 | 8 | c-terminal |  | early onset | LF |  |  | Netherlands | Kim Cryns et al.2003 |
| 6 | A | AAGCACCGTGCAT | 2605_2606insAGCACCGTGCAT | ins 12 bp codon 869 |  |  | AD | childhood (<18) |  | mild to moderate | Likely Pathogenic | United States | A Eliot Shearer et al.2013 |
| 7 | ACCGTGCATGGCG | A | 2614_2625delCATGGCGCCGTG | del 12 bp codon 872 |  |  |  |  |  |  |  | Switzerland | Nicolas Gürtler er al.2017 |
| 8 | #NA | #NA | #NA | deletion exon 1-8 | 8 |  |  | 15~20 |  | mild to moderate |  | Romania | Irina Resmerita et al.2020 |

AD: autosomal dominant; LF: low frequency; HF: high frequency; VUS: variant of unknown clinical significance.
